# A septate uterus with double cervix during two pregnancies: pregnancy outcome before and after cervix sparing metroplasty. A case report

**Published:** 2020-08-05

**Authors:** EB Seljeflot, ØE Nytun, SB Kjøtrød, M Gergolet

**Affiliations:** Departement of Obstetrics and Gynaecology, St.Olavs hospital, Trondheim University Hospital, Norway;; General Hospital Dr Franc Derganc Nova Gorica, Padlih borcev 13, 5000 Šempeter pri Gorici, Slovenia.

**Keywords:** Cervix sparing metroplasty, Congenital uterine anomaly, Hysteroscopy, Premature delivery, 3D ultrasound

## Abstract

In this case report we present a patient with different pregnancy outcomes after IVF treatments. After an extremely premature delivery in her first pregnancy, a vaginal 3D ultrasound scan revealed a correct diagnosis for her uterine anomaly: A complete uterine septum, double cervix and non-obstructive longitudinal vaginal septum, classified as U2bC2V1 according to the European Society of Human Reproduction and EmbryologyEuropean Society for Gynaecological Endoscopy (ESHRE-ESGE)-classification. She underwent a cervix sparing metroplasty and had a spontaneous vaginal birth at 41+2 weeks gesttation. For patients with otherwise unexplained infertility or earlier premature delivery, a diagnostic workup, oriented to the research of uterine malformations, should be taken into consideration.

## Introduction

Approximately 10% (Vander Borght and Wyns, 2018) of child-wanting couples need some kind of medical treatment. According to European Society of Human Reproduction and Embryology (ESHRE) reports, more than 8 million children are born as a result of assisted reproductive technology (ART) since the first baby was born in 1978. As ART becomes more available and success rates improve, the role of reproductive surgery seems to be neglected (Raff and DeCherney, 2019). For some infertile couples ART may not be the only option to achieve a successful pregnancy, or even worse, may not be sufficient as a strategy on its own. Reproductive surgery should be considered if pathologies of the uterus or fallopian tubes are diagnosed. In this case report we show how hysteroscopic cervix sparing metroplasty for a complete uterine septum and double cervix affected the outcomes of a woman with three pregnancies after in vitro fertilisation (IVF).

## Case Presentation

### 

A 32-year-old nulligravida was referred for infertility treatment after three years of attempts.Previously, she had already been diagnosed with a “complete uterus didelphys” by a standard 2D vaginal ultrasound scan and pelvic magnetic resonance imaging (MRI). The MRI described an anomaly with two separate uterine cavities, two cervices and a complete vaginal septum. No further malformations of the urogenital system were found. At the time, the vaginal septum was surgically removed due to dyspareunia. Her monozygotic twin sister was also diagnosed with the same genital malformation.

Due to long-term infertility and a coexisting male factor, the couple underwent ART and achieved pregnancy in the right uterine cavity after a fresh single embryo transfer of a cleavage stage embryo. At 27+6 weeks gestation the patient experienced a preterm premature rupture of the membranes (PPROM) with contractions. She gave birth to a preterm, but otherwise healthy, baby boy.

Two years later, she experienced a miscarriage at the 8 th gestational week after frozen/thawed embryo transfer (FET). A subsequent vaginal 3D ultrasound scan gave a detailed anatomical description of the uterus and cervices, and the genital malformation was reclassified from previous diagnosis of a bicorporal uterus (U3cC2V1), to a complete septate uterus (U2bC2V1) with a double cervix and a longitudinal non obstructive vaginal septum, according to the European Society of Human Reproduction and Embryology-European Society for Gynaecological Endoscopy (ESHRE/ESGE) classification system (Grimbizis et al., 2013). This finding caused a curious discussion among colleagues, before surgical correction prior to further FET was decided.

### The technique:

**Figure 1:**
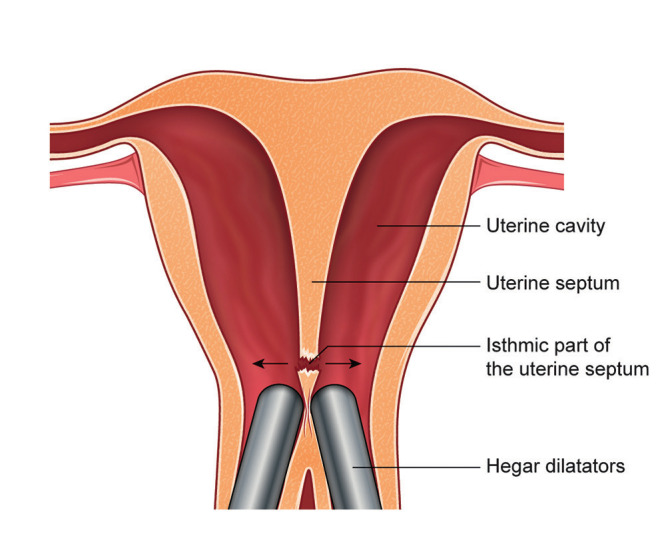
— The cervix sparing technique: The friction between the Hegar dilatators creates rupture of the thinner part of the septum in the isthmical part of the uterus.

In order to create an interruption of the uterine septum without the necessity of unifying the two cervices, we started with the dilatation of both cervices with Hegar dilatators from Hegar number 4 to 12. After inserting a Hegar dilators in one cervix we left it inside and inserted another into the second cervix. In this way we created friction between the two inserted Hegar dilators, especially with the larger dilators. The friction created rupture of the thinner part of the septum which is in the isthmic part of the uterus (Figure 1). Septum resection was continued using a resectoscope through one of the two cervical ostia until the muscular layer was reached. An anti-adhesion gel was inserted as adhesion prophylaxis.

**Figure 2:**
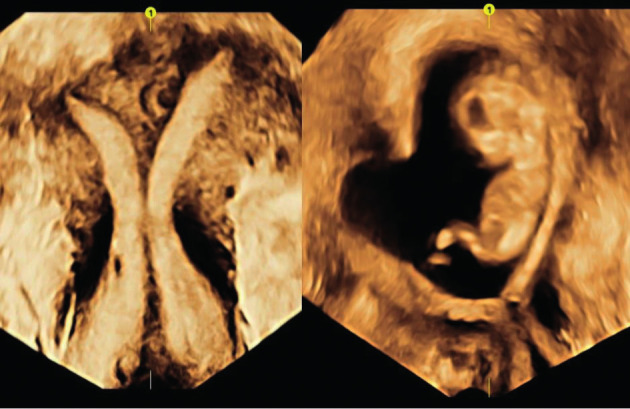
— Left: The uterus before the cervix sparing metroplasty; with a normal outline, a complete septum and two cervices, classified as U2bC2V1 according to the ESGE/ ESHRE-classification. Right: In week 12 of pregnancy the embryo develops in a normal sized uterine cavity, only a small residual septum is seen. The two cervices remain complete.

Two months later a follow up vaginal 3D ultrasound scan showed a uterus with a normal uterine cavity and two cervices. The endometrium was three-laminar and there were no signs of intrauterine adhesions. She achieved pregnancy after FET of a blastocyst five days after ovulation (Figure 2). The luteal phase was supported by standard vaginal progesterone for two weeks. At gestational week 18 she had an ultrasound scan which revealed a male foetus with left renal genesis, a normal right kidney and bladder. She was given vaginal progesterone 100 mg/day as per the department guidelines due to the history of a premature birth. At the 24 th and the 28 th week of gestation both cervices were measured > 3,0cm and the pregnancy was completed without complications. A healthy baby boy was delivered by spontaneous vaginal birth at 41+2 weeks gestation.

## Discussion

A complete septate uterus is associated with infertility, risk of miscarriages, premature birth and fetal malpresentations (Chan et al., 2011). There is no clear evidence supporting the fact that the surgical removal of a uterine septum will reduce the risk of preterm labour, (Rikken et al., 2018) despite numerous studies confirming a possible negative effect of congenital uterine anomalies in pregnancy outcomes (Chan et al., 2011; Venetis et al., 2014). However, there is no data to suggest that metroplasty could affect the subsequent pregnancies negatively (Kenda Suster and Gergolet 2016; Ono et al., 2019).

In cases with a septate or double normal cervix the choice lies between unifying the cervices, with a higher risk for cervical incompetence, or leaving the septation between the two cervical channels. There are a few studies describing the management and reproductive outcome of surgical correction of a complete septate uterus with a double cervix, most of them before the ESHRE/ESGE classification system was published. Without a clear classification, a “double cervix” could include both a septate cervix (C1) and a double “normal” cervix (C2). This may be the reason why some authors recommend cervix sparing metroplasty for patients with a history of poor reproductive outcomes (Chen et al., 2013), while others find the resection of the cervical septum less complicated than a cervix sparing technique and recommend cervical septum resection (Parsanezhad et al., 2006). Studies finding no differences in reproductive outcomes comparing the two groups which underwent metroplasty with or without a cervix sparing technique, do also describe opening of the cervical septum with Metzenbaum scissors (Parsanezhad et al., 2006). Cutting a double normal cervix (C2) with scissors may cause major intraoperative bleeding, scarring and/or a large cervical canal which may cause an iatrogenic cervical incompetence. We found no studies describing the reproductive outcomes after surgery for unifying two normal cervices.

The ESHRE/ESGE classification system, published in 2013 (Grimbizis et al., 2013), provides a description and classification of not only uterine anomalies but also anomalies of the cervix and vagina. The classification presents an absolute novelty, which is class 0, a normal uterus. This is a major improvement, giving the opportunity to combine uterine, cervical and vaginal morphology. A clear classification is able to describe complex anomalies, which is an essential foundation for further research as well as discussion between colleagues.

Our patient experienced an extreme premature birth of her first child, and apart from the uterine malformation she had no other risk factors of premature birth. Considering the numerous publications reporting improvement of pregnancy outcomes after septum resection, metroplasty was proposed to the patient. A major concern was the risk of iatrogenic cervical incompetence, so we performed a cervix sparing metroplasty. Progesterone therapy from gestational week 18 was given per department guidelines in order to reduce the risk of premature labour. However, the effect of this therapy is still uncertain (Norman et al., 2018).

A combined hysteroscopy and laparoscopy procedure is considered the gold standard for diagnosing Müllerian anomalies. Vaginal 2D ultrasound scans have an accuracy of 87%, while 3D scans reveal up to 100% of the anomalies in experienced hands (Grimbizis et al., 2016). Even MRI will not give the correct subclassification of uterine anomalies in all patients. Furthermore, MRI is an expensive procedure, often with long waiting times. On the contrary, vaginal 3D ultrasound scan is a safe, quick, and sensitive way of detecting uterine anomalies (Kougioumtsidou et al., 2019). More than laparoscopy and hysteroscopy, 3D ultrasound gives information not only on the external contour and cavity morphology, but also of myometrial wall thickness (Kougioumtsidou et al., 2019). A good preoperative ultrasound scan will help plan the hysteroscopy procedure, regarding the choice of hysteroscope, anaesthesia and surgical technique. Peroperative ultrasound is also useful during septum resection to avoid uterine wall perforation, especially when treating a bicorporal septate uterus (U3c). The metroplasty can be performed safely in an outpatient setting without the need of an assisted laparoscopic procedure.

The early follicular phase is often the chosen time for the hysteroscopic procedure in order to have a thin endometrial lining and an optimal view. However, for practical and logistical reasons this may involve treating the patient with GnRH agonists, danazol or progestins before surgery to thin the endometrium, which may reduce the operating time, blood loss and fluid absorption (Colacurci et al. 1998; Parazzini et al., 1998). On the other hand, the hypoestrogenic environment could potentially increase the risk of postoperative intrauterine adhesions. Despite decades of research, there is still insufficient evidence for or against recommending preoperative treatment to thin the endometrium prior to hysteroscopic septum resection (Practice Committee of the American Society for Reproductive Medicine 2016).

Both the patient’s monozygotic twin sister and her second child have been diagnosed with urogenital malformations. This raises the question of a genetically predisposed risk, even though most urogenital malformations occur spontaneously. This issue is referred for review by the Department of Medical Genetics.

In this case, the patient is her own control, with completely different outcomes of her pregnancies before and after the surgical correction of her Müllerian anomaly. She has expressed her gratitude for the performed metroplasty, but also her concerns for other women who might not get the same opportunity as she did, due to a lack of knowledge and neglect of reproductive surgery as an alternative or addition to ART.

## Conclusion

We recommend an evaluation of the uterus with a vaginal 3D ultrasound scan as part of the routine examination for all women referred for fertility assessment. A complete diagnosis is essential as uterine malformations may reduce the success rate of ART, and the detection of correctable malformations could spare patients from unnecessary burdens e.g. miscarriages, preterm births and possible ART as well. The technique described here is safe, easy to perform, an leaves the cervix unmodified without an abdominal approach.
